# Spectral cardiac CT in acute stroke patients

**DOI:** 10.1038/s41598-023-33940-3

**Published:** 2023-04-25

**Authors:** Naomi Larsen, Friederike Austein, Tristan Klintz, Graeme Campbell, Sam Sedaghat, Schekeb Aludin, Domagoj Schunk, Marcus Both, Olav Jansen, Patrick Langguth

**Affiliations:** 1 Department of Radiology and Neuroradiology, University Medical Center Schleswig-Holstein, Arnold-Heller-Str. 3, 24105 Kiel, Germany; 2 Department of Diagnostic and Interventional Neuroradiology, University Medical Center Hamburg-Eppendorf, Martinistr. 52, 20246 Hamburg, Germany; 3 Philips Healthcare, Clinical Science, Philips GmbH Market DACH, Röntgenstr. 22, 22335 Hamburg, Germany; 4 Interdisciplinary Emergency Department, University Medical Center Schleswig-Holstein, Arnold-Heller-Str. 3, 24105 Kiel, Germany

**Keywords:** Computed tomography, Stroke

## Abstract

Cardiac CT obtained in acute ischemic stroke patients can facilitate timely detection of cardiac sources of embolism and guide secondary prevention strategies. Spectral CT exploiting the simultaneous acquisition of separate higher-energy and lower-energy photon spectrum datasets has the potential to improve contrast between thrombi and cardiac structures. This study aimed to investigate the diagnostic value of spectral cardiac CT compared to conventional CT for the detection of cardiac thrombi in acute stroke patients. Patients with acute ischemic stroke undergoing spectral cardiac CT were retrospectively included. Conventional CT images, virtual 55 keV monoenergetic (monoE55), z-effective (zeff), and iodine density images were evaluated for the presence of thrombi. Diagnostic certainty was rated on a 5-point Likert scale. Contrast ratios were calculated for all reconstructions. 63 patients with 20 thrombi were included. Four thrombi were missed on conventional images but detected on spectral reconstructions. MonoE55 achieved the highest scores for diagnostic certainty. Contrast ratios were highest on iodine density images, followed by monoE55, conventional and zeff (*p* < 0.005). Spectral cardiac CT adds diagnostic benefit for the detection of intra-cardiac thrombi in acute ischemic stroke patients compared to conventional CT.

## Introduction

Twenty percent of ischemic strokes can be attributed to a cardiac source of embolism^1^. Cardiac imaging can detect cardioembolic sources, with transthoracic (TTE) and transesophageal echocardiography (TEE) being the most widely used methods in stroke patients^2^.

Cardiac CT is able to detect intra-cardiac thrombi and minor sources of cardio-embolism, such as mitral valve calcifications, with high sensitivity and specificity^3–5^. In acute stroke patients, cardiac CT can be performed as part of a one-stop shop stroke CT protocol for the immediate detection of cardioembolic sources^6^.

Dual-layer spectral CT exploits the simultaneous acquisition of separate higher-energy and lower-energy photon spectrum datasets allowing for material decomposition. Specifically, virtual low-keV monoenergetic and iodine density images have been shown to improve contrast between iodine and soft tissue, and contrast-to-noise ratio^7,8^. Z-effective (zeff) images can be used to differentiate between tissue components by displaying effective atomic numbers^9^.The method can enhance contrast between iodinated contrast agent, left atrial and ventricular walls, and clots^10–12^, and thus aid in the detection of intra-cardiac thrombi.

This study aimed to determine the additional diagnostic value of spectral CT reconstructions compared to conventional CT for the detection of intra-cardiac thrombi in acute stroke patients.

## Results

### Patients

For characteristics of the included patients, see Table 1.

**Table 1:** Patients’ characteristics.

Parameter	
Female (n/%)	36/57
Age [years] (mean/range)	80/56–96
Heart rate [beats per minute] (mean)	84
Site of occlusion (n/%)	
Middle cerebral artery	49/78
Posterior cerebral artery	5/8
Internal carotid artery	4/6
Anterior cerebral artery	2/3
Basilar artery	2/3
Vertebral artery	1/2
Transthoracic echocardiography*	38/60
Transesophageal echocardiography*	8/13
Time between CT and transesophageal echocardiography [days] (mean)	3.3
Atrial fibrillation (n/%)	33/52
Sinoatrial block (n/%)	1/2
Dilated cardiomyopathy	1/2

*Within 10 days after cardiac CT.

### Radiation dose

The mean dose length product (DLP) for the complete multimodal CT scan including delayed-phase cardiac CT was 1834 mGy cm. The mean value for the cardiac scan alone was 296 mGy cm.

### Contrast

**Figure 1:**
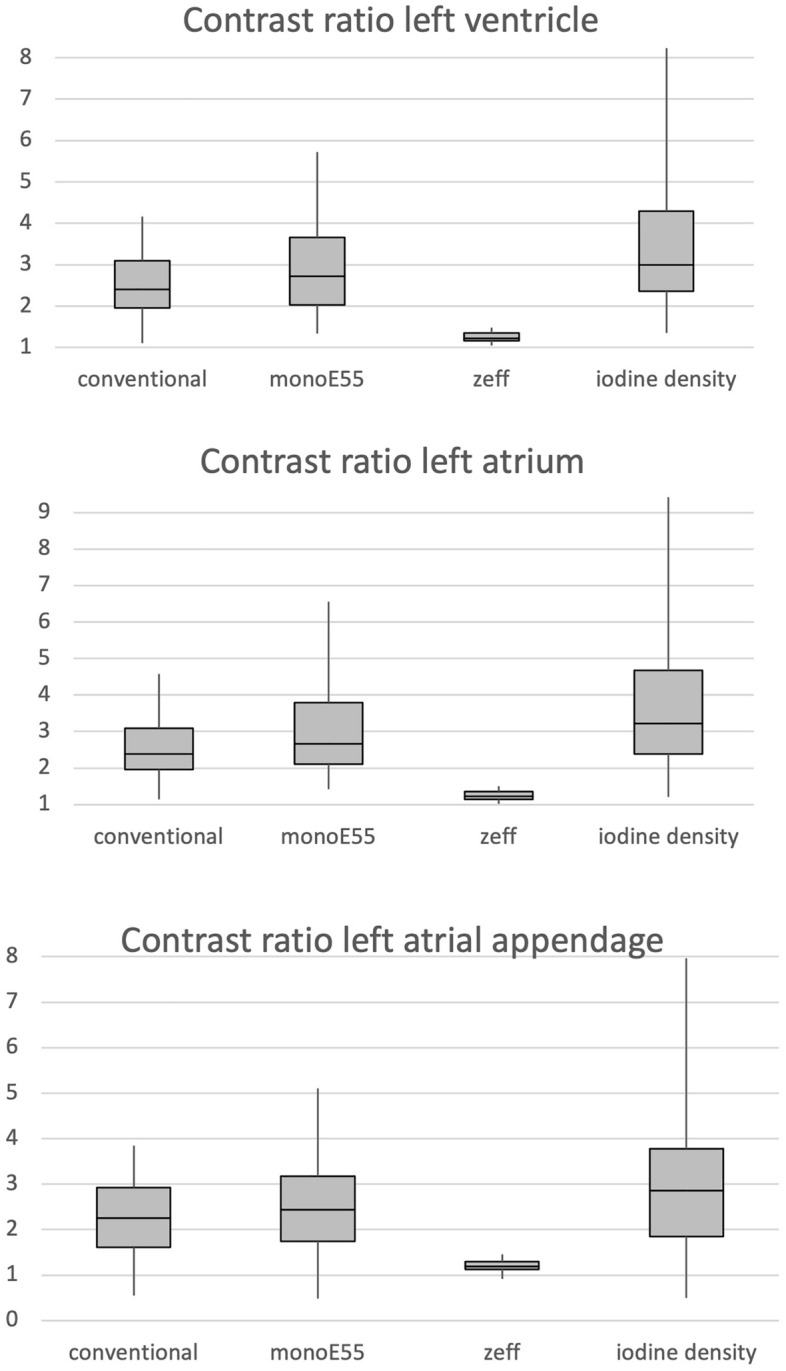
Box plot distribution of contrast ratios of the left ventricle (top), left atrium _(_middle), and left atrial appendage (bottom) for conventional, virtual 55 keV monoenergetic (monoE55), z-effective (zeff), and iodine density reconstructions. MonoE55 and iodine density images achieved higher contrast ratios in all localizations compared with conventional images (*p* < 0.005).

Contrast ratios in the left ventricle (LV), left atrium (LA) and left atrial appendage (LAA) were highest on iodine density images, followed by virtual 55 keV monoenergetic (monoE55), conventional and zeff (*p* < 0.005) (Fig. 1).

### Major cardioembolic sources

**Figure 2:**
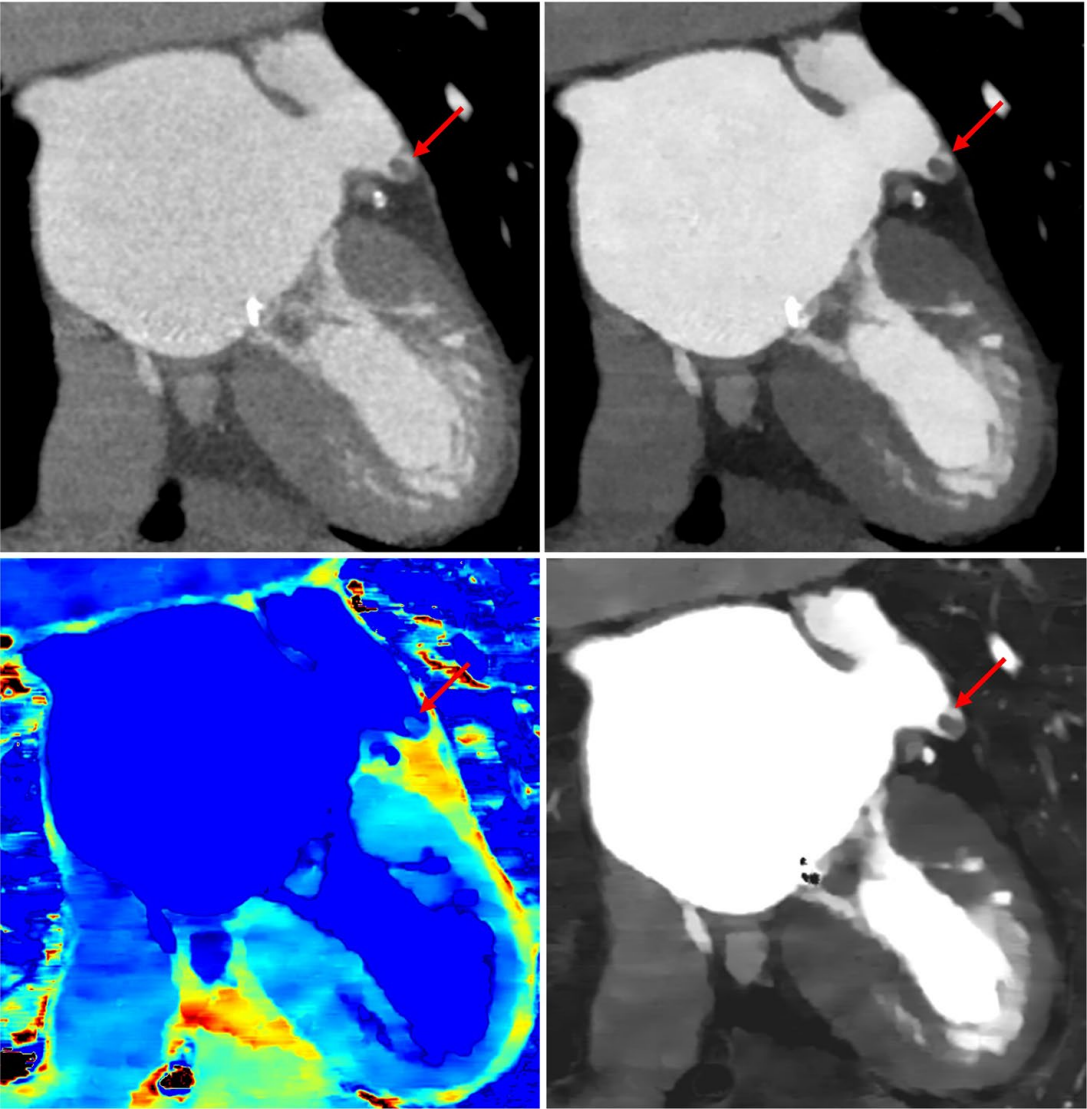
5 mm thrombus in the left atrial appendage (arrows) on conventional (top left), virtual 55 keV monoenergetic (top right), z-effective (bottom left), and iodine density images (bottom right).

**Figure 3:**
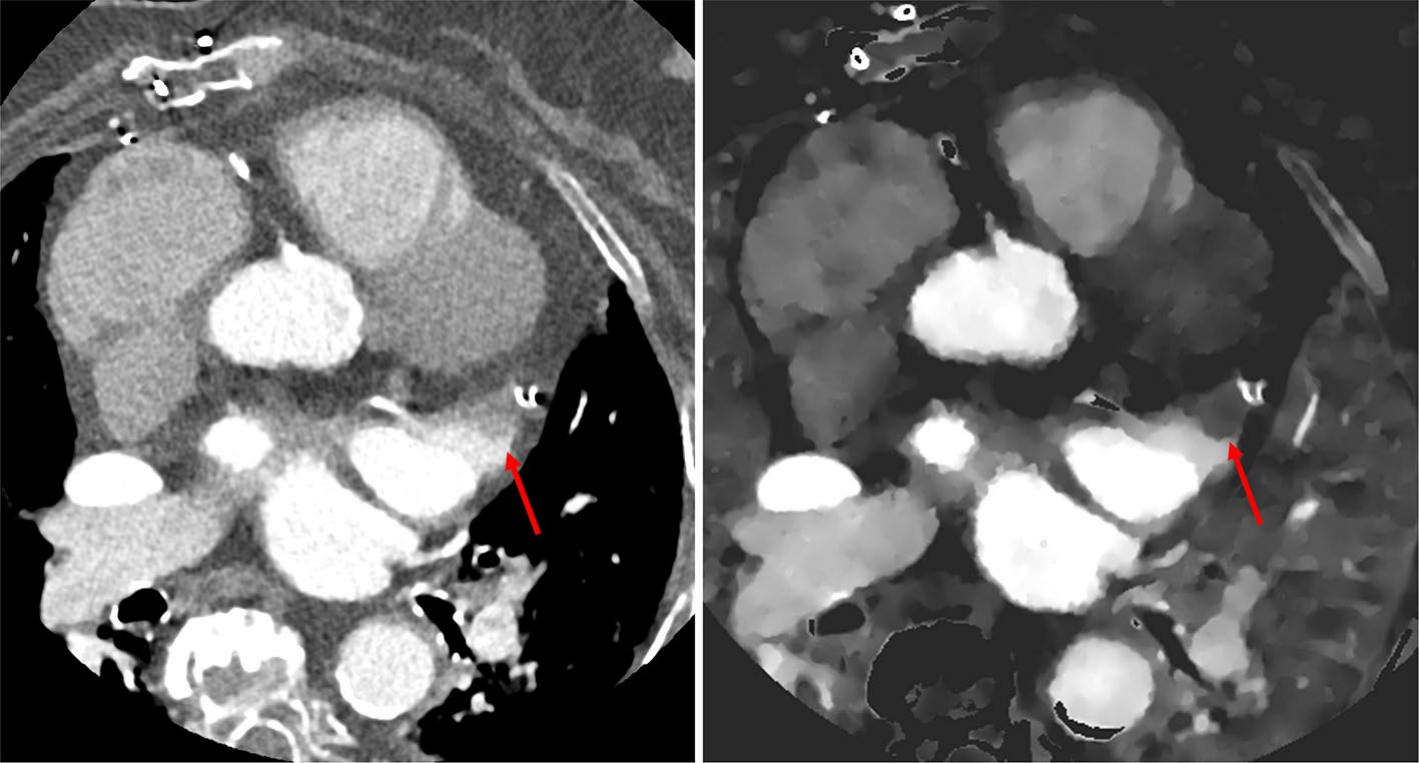
9 mm thrombus in the left atrial appendage, which was missed on conventional cardiac CT images (left). The finding was classified as circulatory stasis. The thrombus was diagnosed on iodine density reconstructions (right).

Twenty intra-cardiac thrombi were detected in 15 patients (1 thrombus in 11 patients, 2 thrombi in 3 patients, 3 thrombi in 1 patient). Cohen’s kappa coefficient reached values of 0.477 indicating a moderate interrater agreement for the diagnosis of thrombi on conventional images, 0.580 for monoE55, 0.308 for zeff, and 0.3.75 for iodine density reconstructions, respectively. Illustrative cases are displayed in Fig. 2 and 3.

Mean thrombus length was 11 mm (range, 4–24 mm). Table 2 summarizes the number of thrombi detected in conventional and spectral image reconstructions depending on location.

**Table 2:** Detection rate of intra-cardiac thrombi in conventional and spectral image reconstruction datasets.

	Intra-cardiac thrombus	Conventional	monoE55	Zeff	Iodine density
Left atrial appendage	9	6	8	6	9
Left ventricle	8	7	8	4	8
Mitral valve*	3	3	3	0	1
N (%)	20 (100)	16 (80)	19 (95)	10 (50)	17 (85)

monoE55, virtual 55 keV monoenergetic; Zeff, z-effective.

*All thrombi were found on prosthetic mitral valves.

Of other major cardioembolic sources, LV aneurysm was detected in 7 cases, hereunder 5, which were depicted on all reconstructions, whereas 2 were missed on zeff reconstructions.

One thrombus was detected by TEE, TTE failed to detect any thrombi. One LAA thrombus was confirmed by autopsy. TEE depicted one LV aneurysm. A prosthetic aortic valve was present in 4 patients, a prosthetic mitral valve in 3 patients.

### Minor cardioembolic sources

Mitral annulus calcifications were present in 12 cases. Persistent foramen ovale (PFO) was detected in 2 patients, 1 of which was not depicted on conventional images, but only on monoE55 and iodine density reconstructions, and was confirmed by TEE.

### Best image reconstructions

MonoE55 reconstruction was rated best in 82% of cases, iodine density in 14%, conventional images in 4%, and zeff in 0%.

### Diagnostic certainty

Association between diagnosis (thrombus yes/no) and Likert scores was strongest for monoE reconstructions (Spearman’s rho 0.784), followed by conventional images (0.750), iodine density (0.705) and zeff (0.451).

## Discussion

Patients with ischemic stroke related to cardio-embolism have a poorer prognosis compared to other stroke subtypes, including higher mortality and increased risk of stroke recurrence^13^. Moreover, in patients with stroke of undetermined etiology, LAA thrombus has been described as the most prevalent among major cardioembolic sources^14^. This study aimed to determine the additional value of spectral reconstructions for the detection of cardio-embolic sources in the setting of a one-stop shop stroke CT protocol including delayed-phase spectral cardiac CT-imaging.

Four intra-cardiac thrombi (20%) would have been missed if only conventional images were available. Virtual monoenergetic images appeared to be the most valuable reconstruction with nearly all thrombi being depicted (5% missed), diagnostic certainty for the presence or absence of thrombus being highest, and virtual monoenergetic images ranking highest in the subjective ranking among all reconstructions.

Contrast and thereby conspicuity of thrombi in the LAA, LA and LV was enhanced using spectral reconstructions, with the highest contrast ratio in iodine density reconstructions, followed by monoE55, which is in line with previously conducted phantom studies^15^. Accordingly, one thrombus in the LAA was detected on iodine density images only. On the other hand, iodine density images failed to depict thrombi at the mitral valve. Possibly, iodine concentration measurements are less reliable in the presence of motion artifacts that are more prevalent in the LV and at the valves compared to the more stationary LAA cavity, especially in this emergency cardiac CT protocol without administration of beta-blockers for heart-rate control.

The value of imaging intra-cardiac thrombi in the presence of atrial fibrillation has been the subject of debate^2,14,16^. Still, emergent cardiac CT imaging in acute stroke could be beneficial due to the direct detection of intra-cardiac thrombi in close temporal proximity to the event compared to echocardiography, which is often delayed by several days in clinical practice, and might fail to demonstrate thrombus due to thrombolytic processes^6^. Particularly TEE can frequently not be performed in acute stroke patients due to its semi-invasive nature, the need for sedation and the prevalence of relative contraindications as dysphagia^17^.

An additional value of spectral images was also noted in this study for the detection of minor cardioembolic sources such as PFO, which is relevant since identification of PFO can have an implication for secondary prevention therapy in a subset of stroke patients^18–20^.

The major limitation of this study is the retrospective study design and small study population including 63 patients, which did not allow for subgroup analyses. The lack of a reference standard for the diagnosis of intra-cardiac thrombi in a sufficient number of patients precluded calculation of diagnostic measures such as sensitivity and specificity. Still, cardiac CT is an established method for the detection of stroke-related pathologies and has been reported to achieve an even higher sensitivity in the detection of thrombi in the LA, LAA and LV in a single comprehensive examination than stand-alone TTE or TEE^21,22^. The focus of this study was to specifically evaluate the additional value of spectral CT imaging compared to conventional CT in an explorative manner.

Moreover, an increase in radiation exposure and application of contrast agent introduced by the cardiac CT poses a potential additional risk to the patient. However, cardiac CT offers a diagnostic advantage in the early diagnosis of cardioembolic causes allowing for timely secondary prevention therapy. The risk of radiation-induced disease is likely less relevant in this relatively old patient cohort and, in our opinion, does not outweigh the potential benefit.

## Conclusions

Spectral imaging adds diagnostic benefit to emergency cardiac CT in stroke patients. Specifically, virtual monoenergetic and iodine density images can aid in the detection of major and minor cardioembolic sources that are missed with conventional imaging alone.

## Material and methods

The ethics committee of the Medical Faculty at Christian-Albrechts-University Kiel approved this study. The research involved in this study was conducted in accordance with relevant guidelines and regulations, and the Declaration of Helsinki. Informed consent was obtained from all patients. We retrospectively identified all acute stroke patients with a thrombotic occlusion of the ICA, MCA (M1 and M2 segments), ACA (A1 and A2 segments), the V4 segments of the vertebral artery, the basilar artery, or PCA (P1 and P2 segments), who underwent a delayed-phase cardiac spectral CT as part of a multimodal CT protocol for acute stroke patients between April 2019 and February 2020 in our institution, a tertiary care hospital with stroke unit. All patients were scanned with a dual-layer spectral CT scanner (IQon Spectral CT, Philips Healthcare, Best, The Netherlands).

The multimodal CT protocol comprises non-contrast-enhanced brain CT, non-electrocardiography (ECG)-gated CTA ascending from the origin of the aortic arch to the vertex of the head, CT Perfusion of the brain and delayed-phase cardiac CT covering the entire heart, performed during a single breath-hold with retrospective ECG-gating, descending from the origin of the aortic arch to the diaphragm.

Scan parameters were as follows^6^: 64 × 0.625 mm collimation, 0.27 s gantry rotation time, 100 kV or 120 kV tube voltage, and 375 mA tube current.

For CTA, a 40 ml bolus of iodinated contrast media (Imeron 350, Bracco, Milan, Italy) was intravenously administered, followed by 40 ml for CTP, and 45 ml for the cardiac CT. Cardiac imaging was performed immediately after CTA and CTP, injecting the contrast agent at a rate of 5 ml/s.

Conventional cardiac CT images were reconstructed in axial orientation with a slice thickness of 0.9 mm at the 40% RR-interval.

For each cardiac scan, spectral basis image datasets were computed and transferred to a workstation for analysis (IntelliSpace Portal V11.1.6, Philips Healthcare, Best, The Netherlands). MonoE55, Zeff, and iodine density images of the cardiac scan were reconstructed with a slice thickness of 0.9 mm.

### Image analysis

Images were assessed by two independent readers, a board-certified radiologist with 10 years (P.L.), and a board-certified radiologist and neuroradiologist with 6 years (N.L.) experience in cardiac imaging, who were blinded to clinical data. Disagreement concerning major and minor cardioembolic sources was solved in a consensus reading session. For each case, all reconstructions were analyzed in one reading session. The readers were allowed to use multiplanar views, and to compare images from different reconstructions side-by-side. A thrombus was diagnosed when a filling defect with a round or oval shape was visible in any reconstruction in the LAA, LA, or LV. Circulatory stasis in the LAA, a potential thrombus mimic, was defined as a homogeneous filling defect with a linear border on conventional or monoE55 images. If circulatory stasis was suspected, regions of interest (ROIs) were placed in the filling defect and in the ascending aorta (AA) on the same axial conventional image slice and the mean Hounsfield units (HU) ratio filling defect/AA was calculated. A HU ratio > 0.2 confirmed circulatory stasis^10^.

Both readers rated the diagnostic certainty on a 5-point Likert scale (0 = highly unlikely, 1 = unlikely, 3 = equivocal, 4 = likely, 5 = highly likely) for each reconstruction dataset. The presence of other major sources of cardio-embolism (LV aneurysm, cardiomyopathies, intra-cardiac tumors, valve vegetations), and minor sources (mitral valve calcification, PFO) was documented. Each reader subjectively determined the best reconstruction in each case.

To quantitatively assess and compare contrast between the lumen of the heart cavities and soft tissue in the different reconstruction datasets, contrast ratios were calculated. To obtain the values (HU in conventional and monoE55, absolute numbers in zeff, and mg/ml in iodine density images), a circular region of interest was placed by one reader (P.L.) in the lumen of the LV, LA, and LAA, respectively. The maximum ROI size that was possible to draw without including wall, papillary or pectinate muscles, or valves, was chosen. Accordingly, circular ROIs exclusively including pixels containing papillary muscle tissue were created. Measurements were conducted in each reconstruction dataset. Contrast ratios were calculated as follows:

$${\text{contrast}}\;{\text{ratio}}_{{{\text{LV}}}} = \frac{{mean\; units\; ROI\;\left( {LV} \right)}}{{mean \;units\; ROI\;\left( {papillary \;muscle} \right)}}$$

$${\text{contrast}}\;{\text{ratio}}_{{{\text{LA}}}} = \frac{{mean \;units\; ROI\;\left( {LA} \right)}}{{mean \;units\; ROI\;\left( {papillary\; muscle} \right)}}$$

$${\text{contrast}}\;{\text{ratio}}_{{{\text{LAA}}}} = \frac{{mean \;units \;ROI\;\left( {LAA} \right)}}{{mean\; units\; ROI\;\left( {papillary\; muscle} \right)}}$$

### Statistical analysis

Interrater agreement was tested with Cohen’s kappa. Occurrence of major and minor cardioembolic sources are given in absolute numbers and percentage. An association between Likert scores and diagnosis of thrombus was tested with Spearman rank correlation for each reconstruction. Differences between contrast ratios of conventional images, monoE, zeff, and iodine density reconstructions were tested with the Friedman test. Statistical significance was defined as *p* < 0.05.

## Data Availability

The datasets generated during and/or analysed during the current study are available from the corresponding author on reasonable request.
